# mTOR kinase leads to PTEN-loss-induced cellular senescence by phosphorylating p53

**DOI:** 10.1038/s41388-018-0521-8

**Published:** 2018-10-18

**Authors:** Seung Hee Jung, Hyun Jung Hwang, Donghee Kang, Hyun A. Park, Hyung Chul Lee, Daecheol Jeong, Keunwook Lee, Heon Joo Park, Young-Gyu Ko, Jae-Seon Lee

**Affiliations:** 1 Department of Molecular Medicine, College of Medicine, Inha University, Incheon, 22212 Korea; 2 Hypoxia-related Disease Research Center, College of Medicine, Inha University, Incheon, 22212 Korea; 3 Department of Biomedical Science, Hallym University, Chuncheon, Gangwon-do 24252 Korea; 4 Department of Microbiology, College of Medicine, Inha University, Incheon, 22212 Korea; 5 Division of Life Sciences, Korea University, Seoul, 02841 Korea

**Keywords:** Oncogenes, Senescence, Cell signalling

## Abstract

Loss of PTEN, the major negative regulator of the PI3K/AKT pathway induces a cellular senescence as a failsafe mechanism to defend against tumorigenesis, which is called PTEN-loss-induced cellular senescence (PICS). Although many studies have indicated that the mTOR pathway plays a critical role in cellular senescence, the exact functions of mTORC1 and mTORC2 in PICS are not well understood. In this study, we show that mTOR acts as a critical relay molecule downstream of PI3K/AKT and upstream of p53 in PICS. We found that PTEN depletion induces cellular senescence via p53-p21 signaling without triggering DNA damage response. mTOR kinase, a major component of mTORC1 and mTORC2, directly binds p53 and phosphorylates it at serine 15. mTORC1 and mTORC2 compete with MDM2 and increase the stability of p53 to induce cellular senescence via accumulation of the cell cycle inhibitor, p21. In embryonic fibroblasts of PTEN-knockout mice, PTEN deficiency also induces mTORC1 and mTORC2 to bind to p53 instead of MDM2, leading to cellular senescence. These results collectively demonstrate for the first time that mTOR plays a critical role in switching cells from proliferation signaling to senescence signaling via a direct link between the growth-promoting activity of AKT and the growth-suppressing activity of p53.

## Introduction

In proliferating cells, various stresses trigger cellular senescence, which acts as a tumor suppression mechanism [1, 2]. Overexpression of oncogenes (e.g., RAS, BRAF, or MYC) induces cellular senescence (termed oncogene-induced senescence, or OIS) [3, 4]. Multiple lines of evidence indicate that increased PI3K/AKT signaling induces cellular senescence in many cell types [5, 6]. In addition, loss of phosphatase and tensin homolog (PTEN), the major negative regulator of the PI3K/AKT pathway, triggers cellular senescence through a p53-dependent pathway called PTEN-loss-induced cellular senescence (PICS) [7, 8]. Since AKT and PTEN are among the most commonly hyperactivated and inactivated genes in human cancers, respectively [8], PICS and active AKT-induced senescence could potently guard against tumorigenesis. Although RAS- and MYC-induced senescence have been well characterized, we do not yet fully understand the mechanisms underlying the cellular senescence induced by PTEN-loss and AKT activation [4, 9].

The mammalian target of rapamycin (mTOR), which is a serine/threonine kinase that plays central roles in various biological processes [10], is a major component of the protein complexes, mTOR complex 1 (mTORC1) and mTOR complex 2 (mTORC2). mTOR phosphorylates distinct sets of substrates in response to growth factors (GFs), stress, nutrient availability, and other stimuli [11]. Thus, dysregulation of mTOR is a common feature of many diseases, including cancer, obesity, and type 2 diabetes [12]. The binding of GFs to their receptors triggers phosphatidylinositol-3,4,5-triphosphate (PIP_3_) accumulation and the subsequent recruitments of AKT and 3-phosphoinositide-dependent kinase 1 (PDK1). The recruited AKT is phosphorylated at T308 by PDK1 and at S473 by an mTORC2 complex that includes mTOR, Rictor, mLST8, DEPTOR, Protor1/2, and SIN1. Thus activated AKT phosphorylates and inactivates TSC2; this allows Rheb to activate an mTORC1 complex composed of mTOR, Raptor, mLST8, DEPTOR, and PRAS40. mTORC1 phosphorylates the p70 ribosomal protein, S6 kinase (S6K), and eukaryotic translation-initiation factor 4E (eIF4E)-binding protein 1 (4EBP1) to activate protein translation and cell survival [13]. The function and regulation of mTORC2 is less well defined, although it is known to play roles in activating AKT, SGK, and PKC to regulate cell survival and cytoskeletal organization [14–16]. Liu et al. recently found that mTORC2 is negatively regulated via the phosphorylation of Rictor and SIN1. Conversely, Yang et al. demonstrated that SIN1 phosphorylation enhances mTORC2 kinase activity and fully activates AKT. Although the mTOR pathway has been consistently shown to have a critical function in cellular senescence [9, 17, 18], the exact roles of mTORC1 and mTORC2 in PICS are not well known.

Here, we set out identify the critical relay molecule that lies between PI3K/AKT and p53 in PICS, to answer an important open question in the field: How does the proliferation signal, PI3K/AKT, activate p53 to trigger premature senescence? We report for the first time that mTOR kinase directly binds to p53 and phosphorylates it at S15. We prove that such molecular action mechanism of mTOR is indispensable for PICS and active AKT-induced cellular senescence.

## Results

### PTEN depletion induces cellular senescence via the p53-p21 signaling pathway without triggering a DNA damage response

**Fig. 1:**
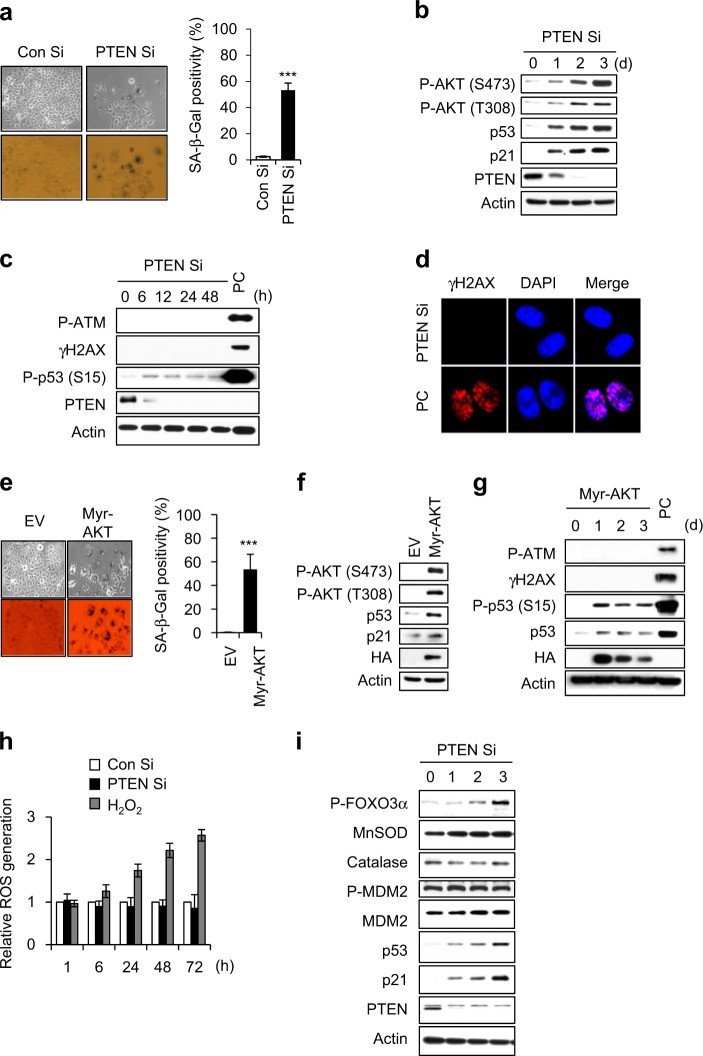
No DNA damage response is involved in the activation of p53-p21 signaling in PICS. **a**–**c** MCF7 cells were transfected with Con Si or PTEN Si. After 4 days, cellular morphology and SA-β-Gal positivity were assessed, and the percentage of senescent cells was quantified (**a**). Western blot (WB) analyses were performed at the indicated times after siRNA transfection (**b**, **c**). **d** Immunocytochemical staining was conducted 1 day after PTEN Si transfection. **e**–**g** MCF7 cells were transfected with a vector encoding HA-tagged Myr-AKT and experiments were performed as described for panels 1**a**–**c**. **h** Analysis of intracellular ROS levels. H_2_O_2_-treated MCF7 cells were used as the positive control. **i** Cells were harvested at the indicated times after Si transfection and then subjected to WB analysis. Cells exposed to 6 Gy IR were used as the positive control (PC) (**b**, **c**). Error bars indicate the ±SD; *n* = 3; ***P < 0.001

We employed siRNA (Si) against PTEN and observed phenotypic and molecular changes (Fig. 1a, b). PTEN-depleted MCF7 cells displayed prematurely senescent phenotypes in a p53/p21-dependent manner (Fig. 1a, b) and showed increased phosphorylation of AKT at serine 473 (S473) and threonine 308 (T308) (Supplementary Fig. S1a–c). As it has been reported that premature senescence is closely correlated with the DNA damage response (DDR) [19, 20], we examined whether DDR is involved in PICS. PTEN depletion did not change the phosphorylation status of the DDR markers, ATM (P-ATM) and H2AX (γH2AX) (Fig. 1c, d). To know whether DDR mediates active AKT-induced senescence (Fig. 1e, f), P-ATM and γH2AX were examined in myristoylated-AKT (Myr-AKT) expressing MCF7 cells. In contrast to IR-exposed positive control (PC) group (Fig. 1c, d, g), P-ATM and γH2AX were not observed in active AKT-induced senescence (Fig. 1g). Active AKT reportedly mediates oxidative stress-induced cellular senescence via FOXO phosphorylation and the subsequent downregulation of MnSOD and catalase [21]. However, we did not observe any change in the levels of intracellular ROS, MnSOD, or catalase during PICS, regardless of FOXO3α phosphorylation (Fig. 1h, i). The ROS scavenger, N-acetylcysteine (NAC), also did not affect SA-β-Gal positivity in cells subjected to PICS or Myr-AKT-induced senescence (Supplementary Fig. S2a–d). These data indicate that DDR and ROS are not involved in PICS and active AKT-induced senescence. We previously reported that depletion of PAPSS2 (3ʹ-phosphoadenosine 5ʹ-phosphosulfate synthetase 2) induced cellular senescence through the FGFR-AKT-p53-p21 signaling pathway [22]. Thus, we examined whether DDR and ROS were involved in a similar type of active AKT-induced senescence (Supplementary Fig. S3a, b). We did not detect any DDR or change in intracellular ROS during the cellular senescence induced by the PAPSS2 depletion-mediated FGFR1-AKT-p53-p21 signaling pathway (Supplementary Fig. S3c–f).

### mTORC1 and mTORC2 are essential for PICS

**Fig. 2:**
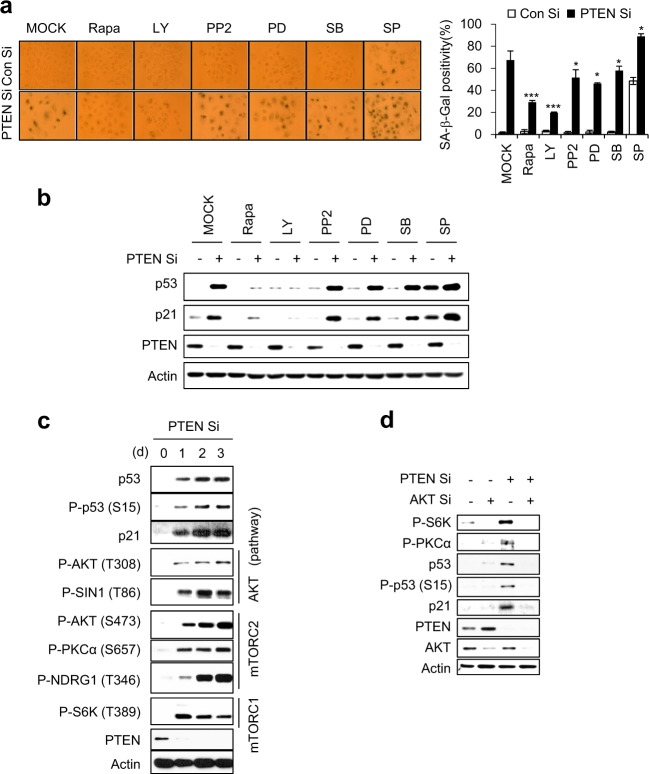
mTORC1 and mTORC2 are activated during PICS. **a**, **b** MCF7 cells were transfected with Con Si or PTEN Si for 6 h, and then treated with the indicated inhibitors. After 4 days, SA-β-Gal positivity was assessed (left panel) and the percentage of senescent cells was quantified (right graph) (**a**). WB analyses were performed 2 days later (**b**). Rapamycin (Rapa), LY294002 (LY), PP2, PD098059 (PD), SB203580 (SB), and SP600125 (SP) inhibit mTORC1, PI3K, Src, Erk, p38, and JNK, respectively. **c** WB analyses were performed at the indicated times after siRNA transfection. **d** MCF cells were transfected with either AKT Si or PTEN Si. After 2 days of transfection, WB analyses were performed. The values represent the mean ± SD; *n* = 3; *P < 0.05; ***P < 0.001

**Fig. 3:**
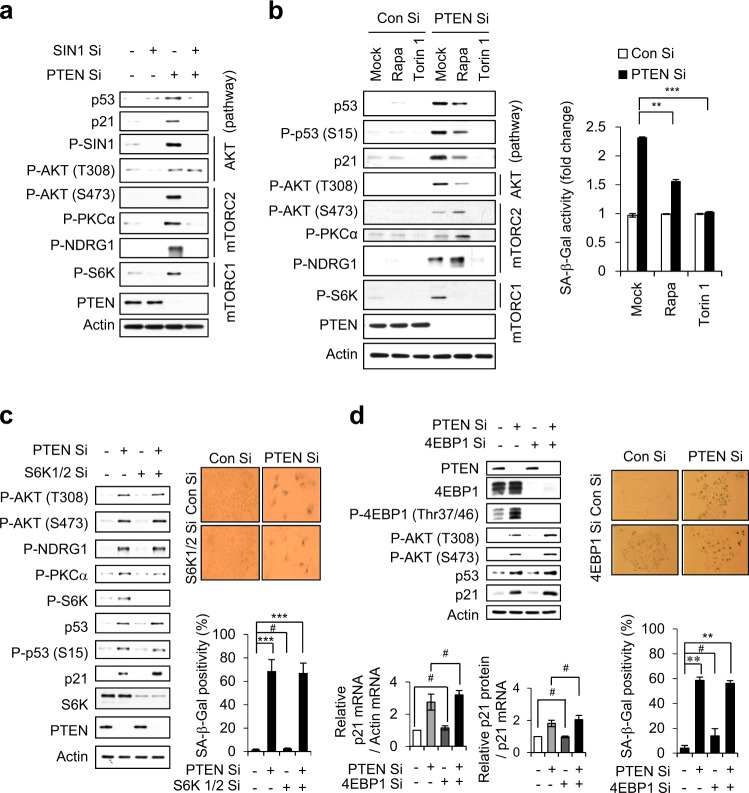
mTORC1 and mTORC2 are critical for PICS, but S6K and 4EBP1 are not. **a** MCF7 cells were transfected with either SIN1 Si or PTEN Si. The cells were harvested at 2 days after transfection for immunoblotting. **b** MCF cells were transfected with Con Si or PTEN Si. After transfection of siRNA, MCF7 cells were incubated with or without Rapa or Torin1. WB analysis and quantification of SA-β-Gal activity were performed after 2 and 4 days, respectively. **c**, **d** All experiments were performed as described in Fig. 3a except using S6K1/2 Si (**c**) 4EBP Si (**d**). WB and qRT-PCR were performed 2 days, and SA-β-Gal activity assays were conducted 4 days after transfection. The values represent the mean ± SD; *n* = 3; ^#^*P* > 0.05; **P < 0.01; ***P < 0.001

To search for the relay molecules that connect active AKT to p53 during PICS, we treated PTEN-depleted cells with six different kinase inhibitors. LY29004- and rapamycin (Rapa)-treated cells showed dramatic decreases in SA-β-Gal positivity and p53/p21 accumulation compared to mock-treated cells (Fig. 2a, b). We examined the phosphorylation of S6K to detect mTORC1 activity [10], and tested the phosphorylations of AKT at S473, PKCα at S657, and NDRG1 at T346 to measure mTORC2 activity [23, 24]. As shown in Fig. 2c, the activities of both mTORC1 and mTORC2 were increased along with that of AKT during PICS. When cells were subjected to double-knockdown with PTEN Si and AKT Si, the phosphorylations of S6K, PKCα, and p53 (at S15) were decreased and p53/p21 accumulation was disrupted (Fig. 2d). Double-knockdown cells transfected with PTEN Si and SIN1 Si, which are downstream targets of T308-phosphorylated AKT [24], showed no phosphorylation of the mTORC1/mTORC2 substrates, S6K, AKT (S473), PKCα, or NDRG1. These double-knockdown cells failed to exhibit the p53/p21 accumulation seen in PTEN Si-treated cells, but phosphorylation of AKT at T308 was still observed (Fig. 3a). These results indicate that mTORC2 is necessary for AKT phosphorylation at S473 and acts upstream of the mTORC1-p53-p21 signaling cascade in PICS.

Rapa specifically inhibits mTORC1, whereas Torin1 inhibits both mTOR complexes. We found that Rapa blocked the PTEN depletion-induced phosphorylation of the mTORC1 substrate, S6K1 (at T389), but partially affected p53/p21 accumulation. In contrast, Torin1 completely prevented p53/p21 accumulation in PTEN-depleted cells (Fig. 3b). Effects of Rapa and Torin were demonstrated in short (0.5 and 1 h) or long (48 h) term treated cells (Fig. 3b and Supplementary Fig. S4). SA-β-Gal positivity was dramatically attenuated in Torin1-treated cells compared to Rapa-treated cells (Fig. 3b). These results indicate that mTORC1 and mTORC2 cooperate with one another to activate p53 in PICS. To further clarify whether S6K activity is involved in PICS, we depleted S6K from PTEN-depleted cells. Cells lacking both S6K and PTEN exhibited levels of p53/p21 accumulation and SA-β-Gal positivity that were comparable to those observed in cells depleted of PTEN alone (Fig. 3c). Cells lacking both 4EBP1 and PTEN also exhibited p53/p21 accumulation and SA-β-Gal positivity at similar levels as in PTEN only depleted cells (Fig. 3d). This indicates that mTORC1 does not contribute to PICS through S6K and 4EBP1 activities, and further suggests that S6K and 4EBP1 activities is dispensable in PICS.

### mTORC1 and mTORC2 physically associate with p53 and directly phosphorylate it at serine 15 during PICS

**Fig. 4:**
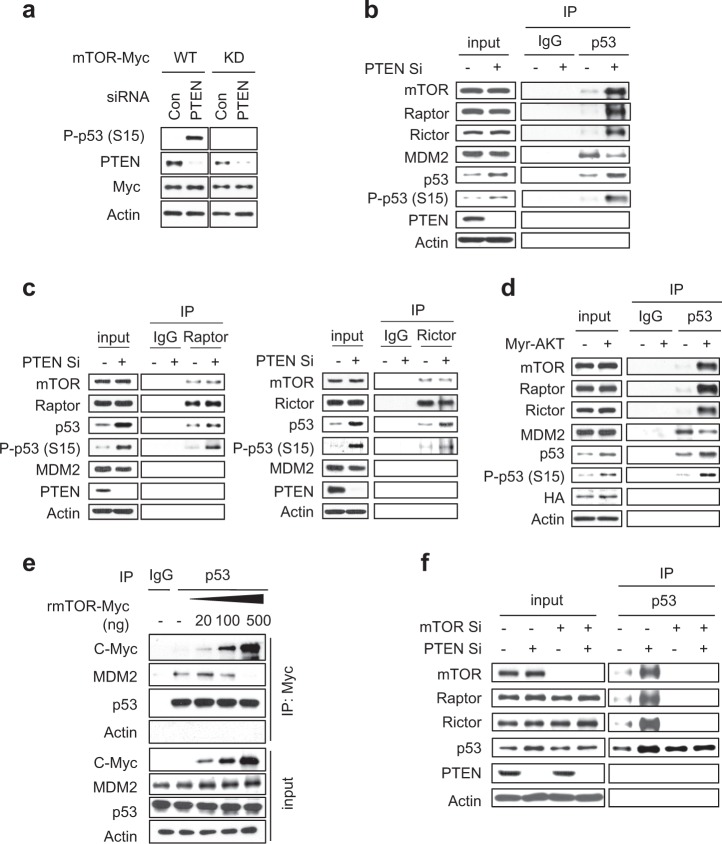
mTORC1 and mTORC2 physically associate with p53 during PICS. **a** MCF7 cells were transfected with vectors encoding Flag-tagged wild-type (WT) mTOR or Flag-tagged kinase-dead (KD) mTOR. The next day, the cells were transfected with the indicated siRNAs. Two days later, WB analyses were conducted. **b**, **d** MCF7 cells were transfected with Con Si, PTEN Si (**b, c**), or Myr-AKT (**d**). After 2 days, the transfected cells were lysed, immunoprecipitated with α-p53, α-Raptor, and α-Rictor antibodies, and immunoblotted with the indicated antibodies. **e** In vitro competition assay. **f** MCF7 cells were transfected with Con Si or mTOR Si. After 6 h, half of each group was transfected with Con Si, and the other half was transfected with PTEN Si. After 2 days, transfected cells were lysed and immunoprecipitated with anti-p53 antibody

We next tested whether mTOR serine/threonine kinase activity in PICS requires that p53 be phosphorylated at S15 and/or accumulated in the cell. We transfected cells with vectors expressing mTOR wild-type (WT) or an mTOR S2035T/D2357E kinase-dead (KD) mutant [25], and observed the phosphorylation of p53 at S15. Indeed, p53 phosphorylation at S15 was abolished in PTEN-depleted cells expressing mTOR KD (Fig. 4a). We further conducted an immunoprecipitation (IP) assay with anti-p53, and observed endogenous association of mTOR with p53 during PICS (Fig. 4b). Raptor and Rictor were also found in the p53 immunoprecipitates, indicating that both mTORC1 and mTORC2 physically associated with p53. Furthermore, p53 was associated with mTORC1 and mTORC2 instead of MDM2 under PTEN-loss condition (Fig. 4b). The physical association of p53 with mTORC1 and mTORC2 and its competition with MDM2 were also observed in Myr-AKT-overexpressing cells (Fig. 4d) and during PAPSS2 depletion-mediated cellular senescence (Supplementary Fig. S5a). The p53 bound to mTORC1 and mTORC2 was the phosphorylated form (Fig. 4b–d). IP assays with anti-Raptor or anti-Rictor antibodies confirmed that both mTORC1 and mTORC2 bound to p53 and phosphorylated p53 at S15 in PICS and PAPSS2 depletion-mediated cellular senescence (Fig. 4c and Supplementary Fig. S5b). To see subcellular localization of mTOR and p53/phospho-p53 (S15), we conducted immunocytochemical analysis. In contrast to control cells, mTOR was co-localized with p53/phospho-p53 (S15) during PICS (Supplementary Fig. S6). Localization of p53/phospho-p53 (S15) with mTOR/Raptor in the lysosome was confirmed by the subcellular fractionation method [26] (Supplementary Fig. S7). We further tested whether MDM2 and mTOR competed with each other for binding to p53 using an in vitro competition assay. As the amount of recombinant mTOR protein (rmTOR) increased, the binding between p53 and MDM2 gradually decreased (Fig. 4e). In mTOR and PTEN double-knockdown cells, the binding of either Raptor or Rictor to p53 was completely inhibited, indicating that the binding of p53 to mTORC1/mTORC2 is mediated by mTOR (Fig. 4f). In mTOR and PAPSS2 double-knockdown cells, the binding of either Raptor or Rictor to p53 was also completely inhibited (Supplementary Fig. S5c).

**Fig. 5:**
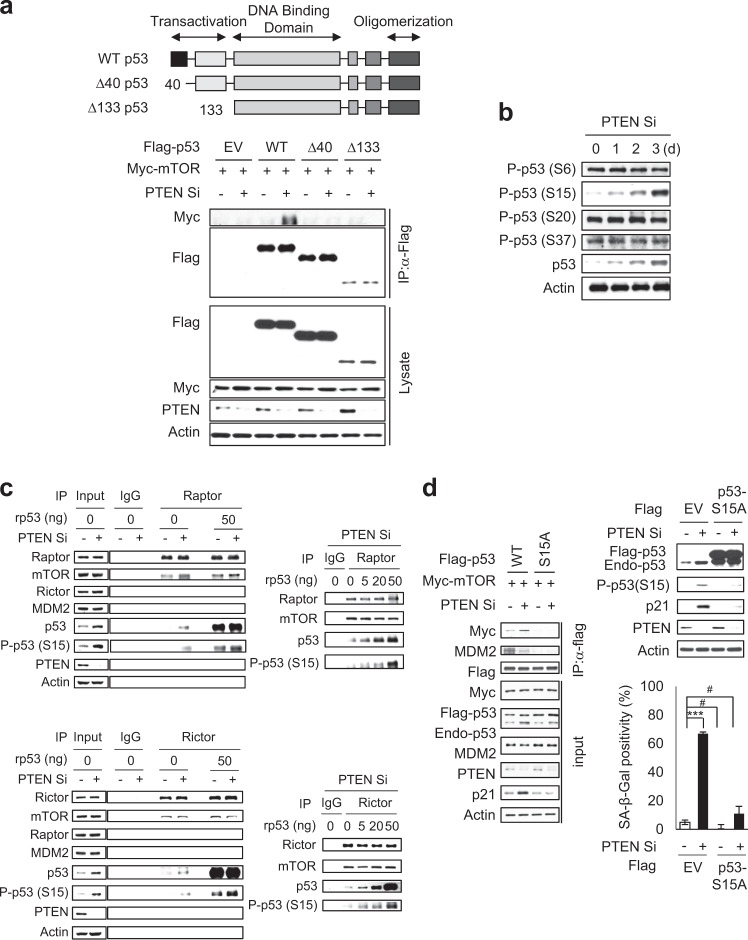
mTORC1 and mTORC2 directly phosphorylate p53 at S15 in PICS. **a** MCF7 cells exogenously expressing flag-tagged WT, Δ40, and Δ133 p53 along with Myc-tagged mTOR were transfected with Con Si or PTEN Si. Two days after siRNA transfection, the cells were immunoprecipitated with anti-Flag antibody. **b** Cells were transfected with Con Si or PTEN Si, harvested at the indicated days, and subjected to immunoblotting. **c** In vitro kinase assay. MCF7 cells were transfected with Con Si or PTEN Si. After 2 days, cell lysates were immunoprecipitated with α-Raptor and α-Rictor antibodies and used for in vitro kinase assay. **d** MCF7 cells exogenously expressing Flag-tagged WT p53 or S15A mutant p53, in combination with Myc-tagged mTOR, were transfected with Con Si or PTEN Si. Two days after siRNA transfection, the cells were immunoprecipitated with anti-Flag antibody. SA-β-Gal activity assays were conducted 4 days after siRNA transfection. The values represent the mean ± SD; *n* = 3; ^#^*P* > 0.05; ***P < 0.001

To identify the domain of p53 that interacts with mTOR, we performed IP assays using p53 WT and the truncated mutants, Δ40 and Δ133 p53, which lack the N-terminal 39 and 132 amino acids, respectively [27]. We found a specific interaction between p53 WT and mTOR but observed that Δ40 and Δ133 failed to bind mTOR (Fig. 5a), indicating that the N-terminal 39 amino acids of p53 are required for its physical association with mTOR. Phosphorylation of p53 at S15 is classically regarded as the first crucial step in the stabilization of p53, as it inhibits the interaction of p53 with MDM2 [28]. When we examined the phosphorylation status of the N-terminal 39 amino acids of p53 in PICS, we found that PTEN depletion specifically induced the phosphorylation of p53 at S15, but not at S6, S20, or S37 (Fig. 5b). To detect the kinase activity of mTOR towards p53, we conducted an in vitro kinase assay using recombinant p53 protein (rp53) and immune-purified mTORC1 and mTORC2. We found that immune-purified mTORC1 and mTORC2 evidently phosphorylated p53 at S15 and its phosphorylation levels were rp53 dose-dependent (Fig. 5c). We observed basal mTORC1 and mTORC2 activities could bind and phosphorylate p53 at S15 to a small extent in the presence of PTEN (Fig. 5c). The faint band in the absence of rp53 (0 ng rp53) shown in in vitro assay with Raptor or Rictor immunoprecipitates was due to co-immunoprecipitation of endogenous p53. In addition, p53 mutants in which S15 was substituted by non-phosphorylatable alanine (S15A) failed to associate with mTOR (Fig. 5d). When p53 S15A was overexpressed, p21 induction was barely detectable and SA-β-Gal positivity was dramatically decreased in PTEN-depleted cells (Fig. 5d). Collectively, these data indicate that mTOR can directly phosphorylate p53 at S15 through physical interactions between p53 and mTORC1 or mTORC2 in the absence of PTEN, finally resulting in PICS.

### Loss of PTEN induces cellular senescence in embryonic fibroblasts of knockout mice

**Fig. 6:**
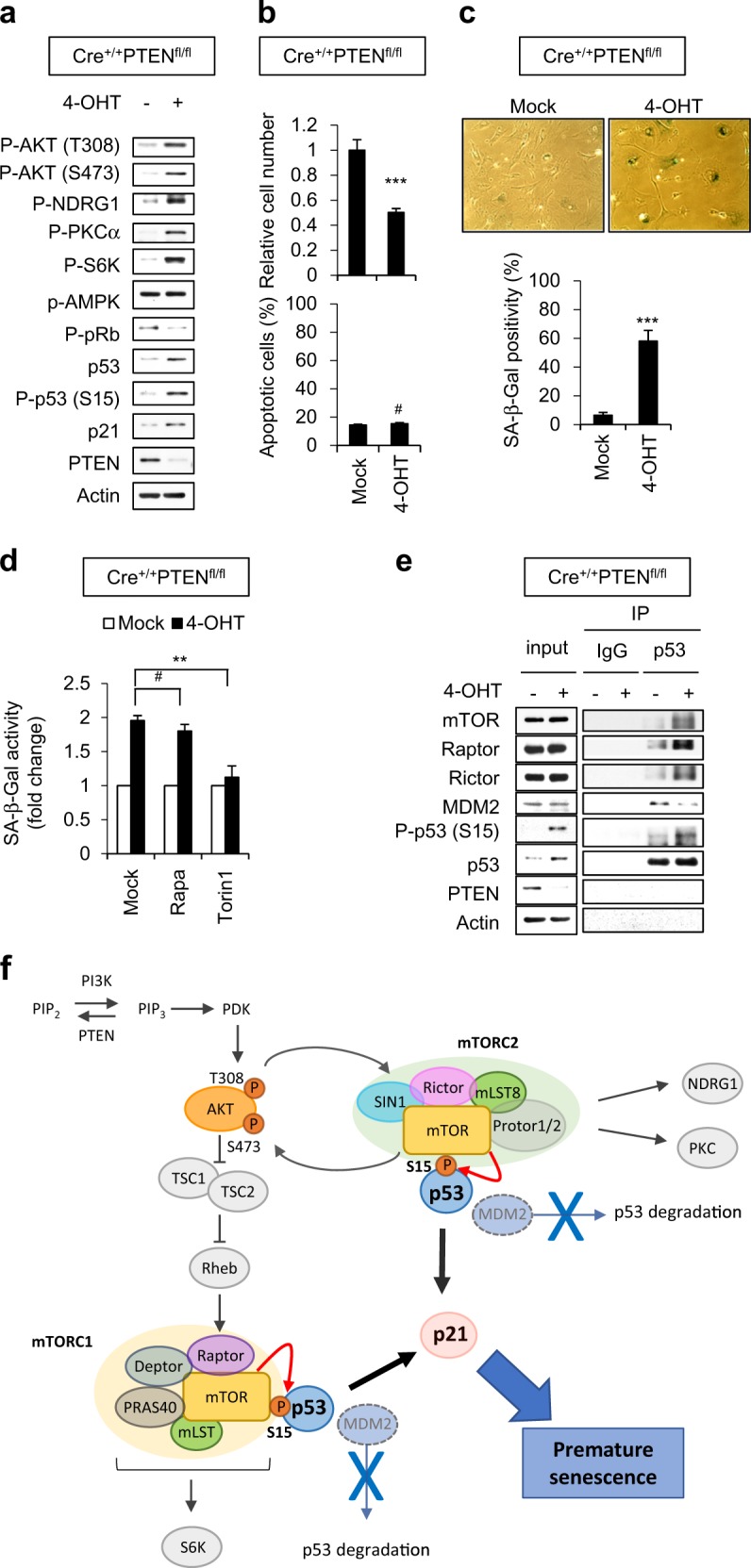
The binding of mTORC1 and mTORC2 to p53 in PTEN-deficient mouse embryonic fibroblasts. **a**–**e** CrePTEN^fl/fl^ MEFs were cultured with or without 0.5 μmol/L 4-OHT for 3 days and subjected to immunoblotting (**a**). Relative cell number (upper graph) and Annexin V positivity (lower graph) were determined at 6 and 4 days, respectively, after 4-OHT treatment (**b**). Morphology and SA-β-Gal positivity were observed at 7 days after 4-OHT treatment (**c**). MEFs were preincubated with Rapa or Torin1 for 1 h, and then cultured with or without 0.5 μmol/L 4-OHT for 7 days. SA-β-Gal activity was determined (**d**). IP was performed 3 days after 4-OHT treatment (**e**). **f** A proposed signaling cascade illustrating the roles of mTORC1 and mTORC2 in directly binding and phosphorylating p53 during PICS. The values represent the mean ± SD; *n* = 3; ^#^*P* > 0.05; **P < 0.01; ***P < 0.001

To explore the biological significance of our findings in vivo, we used mouse embryonic fibroblasts (MEFs) generated by crossbreeding PTEN conditional knockout (KO) (PTEN^fl/fl^) mice with ER-Cre mice to generate PTEN^fl/fl^-ER-Cre mice. When MEFs isolated from PTEN^fl/fl^-ER-Cre mice were treated with 4-OHT, no PTEN protein was detected (Fig. 6a). The phosphorylations of S6K at T389, AKT at S473, NDRG1 at T346, and PKCα at S657 were detected in PTEN KO MEFs, indicating that both mTORC1 and mTORC2 were activated in these cells. Phosphorylation of p53 at S15, loss of phosphorylated pRb, and increased expression of p53/p21 were also observed, along with decreased cell proliferation and increased senescent cells, but no increase in apoptotic cells (Fig. 6b, c). The SA-β-Gal positivity of PTEN KO MEFs was dramatically attenuated in Torin1-treated cells compared to Rapa-treated cells (Fig. 6d). Moreover, we confirmed the endogenous association between p53 and mTOR and its competition with MDM2 in PTEN KO MEFs (Fig. 6e).

Taken together, our results demonstrate that mTORC1 and mTORC2 are critical players in the p53 activation and p21 accumulation of PICS. Mechanistically, mTOR kinase directly associates with p53 and phosphorylates it at S15 in competition with MDM2, leading to premature senescence through the induction of p21 (Fig. 6f).

## Discussion

Ras activation or MYC expression activate DDR to establish OIS. Activation of DDR in OIS is primarily caused by DNA hyper-replication or ROS accumulation. Various studies have shown that DDR plays a central role in the S15 phosphorylation of p53 in cellular senescence [19, 28–30]. It has been reported that AKT activation induces cellular senescence by increasing oxygen consumption and inhibiting the expression of ROS scavengers downstream of FOXO3a [5, 21]. PICS is also p53-dependent and restricts the onset of aggressive cancer through a similar response triggered by active AKT [4, 8]. PICS is associated with enhanced p53 translation in the absence of hyper-proliferation and DDR [7, 17]. The resulting increase in ROS induces DDR and influences p53 activation. Challenging this view, the present study demonstrates that ROS and DDR are not involved in PICS and cellular senescence induced by the PAPSS2 depletion-mediated FGFR1-AKT-p53-p21 signaling pathway (Fig. 1 and Supplementary Figs. S2 and S3).

Our group and others [8, 17] have shown that p53-depleted cells bypass the senescence response in PICS (Supplementary Fig. S1), indicating that p53 is an important potential barrier to PTEN-loss-driven tumorigenesis. Although reciprocal cooperation between PI3K/AKT and p53 has been proposed, its mechanistic relevance has not been well established. The p53-p21 pathways are located in the central senescence program, where they act as effectors of cellular senescence [4]. However, the upstream signaling pathways that link oncogenic stimuli with p53 as a downstream effector in PICS have yet to be fully identified. Here, we uncover the missing link between active AKT and the p53/p21 signaling pathway. Present study indicates that, when proliferation signals are persistently turned on, mTOR acts as a direct upstream kinase of p53 to induce senescence as a failsafe mechanism. We herein show that p53 is directly phosphorylated by mTOR, and that this interaction plays a crucial role in active AKT-induced senescence triggered by various conditions, such as PTEN-loss, the presence of Myr-AKT, and activation of the AKT upstream receptor molecule (Figs. 4–6 and Supplementary Fig. S5).

mTORC1 and mTORC2 are two structurally and functionally distinct mTOR-containing multiprotein complexes, and the functions of these complexes are greatly affected by Raptor and Rictor, respectively [31–33]. Several reports revealed that mTOR signaling plays a critical mTORC1-associated role in the induction of senescence [9, 17, 18]. Attenuation of mTORC1 signaling can delay and reverse replicative and oncogenic RAS-induced senescence [34]. For example, attenuation of mTORC1 signaling can delay or reverse replicative and oncogenic RAS-induced senescence [18], and Rapa treatment converts nutlin-3a-induced senescence into quiescence [18]. Astle et al. showed that PI3K/AKT-induced senescence requires mTORC1-dependent accumulation of p53, and that this occurs via increases in the synthesis and stability (mediated by MDM2 inactivation) of p53. Alimonti et al. [17] reported that p53 accumulation in PICS requires a translational contribution of mTOR. The authors observed that pharmacological inhibition of mTOR by RAD001 markedly decreased SA-β-Gal positive and p53 stained cells. Mechanistically, the activation of mTORC1 downstream of AKT was shown to promote the accumulation of p53 by regulating its translation. However, we observed that there was no change in the levels of MDM2 or phospho-MDM2 during PICS (Fig. 1i). We further found that mTOR competes with MDM2 and stabilizes p53 via its kinase activity in PTEN Si-treated cells and PTEN KO MEFs (Figs. 4–6). Thus, mTOR directly activates p53. This establishes a novel explanation for how sustained AKT activity could switch the cell fate to premature senescence. In addition, this is the first evidence showing that mTORC2, in addition to mTORC1, could mediate cellular senescence signaling. Our results support and expand upon the previous finding that deletion of both p53 and PTEN led to aggressive bladder cancer with upregulation of mTOR [35]. Here, we also show that SIN1 depletion abrogates the phosphorylation of AKT at S473, the activations of mTORC1 and mTORC2, and the accumulation of p53/p21 (Fig. 2). These findings indicate that SIN1 phosphorylation acts positively on mTORC2 function and is required to complete PICS.

In this study, treatment of PTEN-depleted cells with Rapa did not completely revert p53/p21 to control levels and did not completely prevent senescence (Fig. 3b, c). In contrast, SA-β-Gal activity and p53/p21 accumulation were completely abrogated in Torin1-treated cells (Figs. 3 and 6). This confirms that the disruptions of both mTORC1 and mTORC2 more strongly influenced PICS. Reduced expression of mTOR homologs has been associated with an extended lifespan in various model animals, including *C. elegans*, *Drosophila*, and mice [33]. Although mTOR is involved in various important cellular functions, we propose that the decreased cellular senescence seen in response to inhibition of mTOR is largely due to a direct blockade of p53 phosphorylation. It could be separated from mTOR function in protein synthesis mediated by its downstream target, S6K. The hypothesis is supported by our double-knockdown data showing that PICS could occur in the absence of S6K1/2 (Fig. 3d).

In summary, we herein demonstrate that mTORC1 and mTORC2 critically mediate the p53-dependent senescence pathway in the absence of PTEN. Our results suggest that there is an exquisite balance between the growth-promoting activity of AKT and the growth-suppressing activity of p53, and that this balance is critical for preventing both cellular senescence and cancer. Our results could facilitate the rational design of therapeutic strategies targeting tumors with dysregulated PI3K/AKT signaling, and may suggest that mTOR-specific inhibitors could potentially be developed to prevent cellular senescence, ameliorate aging, and treat diabetes, neurodegeneration, and muscle disease.

## Materials and methods

### Cell culture

MCF7 cells were cultured in Dulbecco’s Modified Eagle’s Medium (DMEM; WelGENE, Daegu, Korea) supplemented with 10% fetal bovine serum (FBS; Lonza, Basel, Switzerland), 1% penicillin and streptomycin (WelGENE) at 37 °C in a 5% CO_2_ incubator. Mouse embryonic fibroblasts were cultured in DMEM containing 20% FBS, 1% penicillin and streptomycin, and 1% non-essential amino acid (NEAA).

### Reagents

The biochemical reagents used in this study were as follows: rapamycin (Millipore, Charlottesville, VA, USA), LY294002 (Millipore), PP2 (Millipore), PD098059 (Millipore), SB203580 (Millipore), and SP600125 (Millipore), Torin1 (Selleckchem, Houston, TX, USA), 4-hydroxytamoxifen (4-OHT) (Sigma, St. Louis, MO, USA), human recombinant protein p53 (TP300003) (OriGene, Rockville, MD, USA) and mTOR (TP320457) (OriGene).

### Plasmids

Mammalian expression vectors encoding HA-tagged AKT-CA (constitutively active) were kindly provided by Dr. Yong Nyun Kim (National Cancer Center, Ilsan, Korea). Mammalian expression vectors encoding flag-tagged wild-type (WT) p53, Δ40 p53, and Δ133 p53 were generously provided by JC Bourdon (University of Dundee, Dundee, UK). pcDNA3-Flag mTOR WT (Wild-Type; Addgene plasmid #26603) and pcDNA3-Flag mTOR KD (Kinase Dead; S2035T D2357E; Addgene plasmid #26605) were generous gifts from Jie Chen (University of Illinois, Illinois, USA.). To create Myc-tagged mTOR WT and KD mutant, inserts were released by restriction enzymes from pcDNA3-Flag mTOR WT and mTOR KD, and were inserted to pCMV-Tag3B (Clontech, CA, USA).

### Gene silencing and plasmid transfection

Cells were transfected with 100 nM siRNA duplexes by using of RNAiMAX (Invitrogen, Karlsruhe, Germany). Transfection of plasmids was accomplished using Lipofectamine 2000 reagent (Invitrogen) through the manufacturer’s instructions. The following siRNAs obtained from Bioneer Inc. (Daejeon, Korea) were used in this study:

Con Si (5′-CCUACGCCACCAAUUUCGUdTdT-3′),

PTEN Si (5′-CCACCACAGCUAGAACUUAdTdT-3′)

4EBP1 Si (5′- GACAUAGCCCAGAAGAUAAdTdT-3′)

mTOR Si (5′-AACCCTGCCTTTGTCATGCdTdT-3′)

Raptor Si (5′-CACCTCACTTTATTTCCATdTdT-3′)

Rictor Si (5′-ACUUGUGAAGAAUCGUAUCdTdT-3′)

SIN1 Si (5′-TAGGTACAACAGCAACCA A dTdT-3′)

AKT Si (5′-GACAACCGCCAUCCAGACUdTdT-3′),

AMPKα1 Si (5′-GCAGAAGUAUGUAGAGCA AdTdT-3′),

p53 Si (5′-CACUACAACUACAUGUGUAdTdT-3′),

p21 Si (5′-CUGUACUGUUCUGUGUCCUdTdT-3′),

mouse PTEN Si #1 (5′-CAGGAAUGAACCAUCUACAdTdT-3′), and

mouse PTEN Si #2 (5′-CAGUAGAAAUUGUCCUACAdTdT-3′).

### Western blot and immunoprecipitation

Western blot (WB) and immunoprecipitation (IP) analyses were performed as described previously [22]. The following antibodies were used in this study: Primary antibodies included phospho-AKT at S473 (#4060), phospho-AKT at T308 (#13038), phospho-Foxo3α at Thr32 9 (#9464), phospho-AMPKα1 at Thr172 (#2535), phospho-p70S6K at Thr389 (#9206), phospho-SIN1 at Thr86 (#14716S), phospho-NDRG1 at Thr346 (#5482), phospho-p53 at Ser6 (#9285), Ser9 (#9288), Ser15 (#9284), Ser20 (#9287), and Ser37 (#9289), phospho-pRb at Ser807/811 (#9308), mTOR (#2972), Raptor (#2280), Rictor (#2140), TSC2 (#6935), 4EBP1 (#9452), and phospho-4EBP1 at Thr37/46 (#2855) (Cell Signaling Technology, Danvers, MA, USA); PTEN (#SC-7974), MDM2 (#SC-965), phospho-PKCα at Ser657 (#SC-12356), and p21 (#SC-397) (SantaCruz, Dallas, TX, USA); p53 (#NCL-L-p53-DO7) (DO7, Leica, Milton Keynes, UK); Actin (#G043) (Applied Biological Materials Inc.); Flag (#A8592), and Catalase (#C-0979) (Sigma); Myc (#OP10) (Calbiochem, CA, USA).

### Senescence-associated β-galactosidase staining

Senescence-associated β-galactosidase activity were measured previously described [22]. A cellular senescence live cell analysis assay kit from Enzo Life Sciences were used for quantification of β-galactosidase activity.

### Cell viability, apoptotic cell death, and ROS measurements

Cell viability was assessed by using a trypan blue exclusion assay. Cell suspensions were treated with 0.4% (w/v) trypan blue (GIBCO, grand island, NY, USA) at a 1:1 ratio. Viable (unstained) and dead (stained) cells were counted using a hemocytometer under the microscope. Cell viability was presented as a value relative to the number of Con Si-transfected cells.

The apoptotic cell death was examined using an FITC-annexin V apoptosis detection kit (BD Biosciences, San Diego, CA, USA) as followed the manufacturer’s protocol. Briefly, cells were incubated with 5 μL of FITC-annexin V, added 5 μL of propidium iodide (PI), and then incubated at 37 °C for 30 min in the dark chamber. The stained cells were analyzed using flow cytometry (BD Biosciences).

Levels of ROS was detected through 2’,7’-dichlorofluorescin diacetate (DCF-DA) staining method. Briefly, cells were incubated with 10 mM DCF-DA at 37 °C for 30 min in dark chamber and then analyzed using FACScan flow cytometer with excitation and emission at 490 and 530 nm, respectively [22, 36].

### In vitro kinase assay

In vitro kinase assays were conducted with some modifications as previously described [37]. Immunoprecipitates of Raptor and Rictor were washed three times in low salt wash buffer (50 mM Tris-HCl at pH 7.4, 150 mM NaCl, 1% NP-40), and then washed twice in buffer (25 mM Tris-HCl at pH 7.4, 20 mM potassium chloride). Kinase assays were conducted at 30 °C for 20 min in a final volume of 15 µl kinase buffer (25 mM Tris-HCl at pH 7.4, 50 mM KCl, 10 mM MgCl2, 250 µM ATP) containing indicated concentrations of recombinant p53 (rp53; 0, 5, 20, and 50 ng). Reaction was terminated by the addition of 30 µl of 2 × Laemmli sample buffer. The kinase activity was measured by western blot analysis to detect substrate phosphorylation.

### In vitro competition binding assay

Cell lysates were incubated with the indicated amounts of recombinant mTOR (rmTOR) at 4 °C for 10 min, and then immunoprecipitated with anti-p53 antibody. Immunoblot assays were performed with the indicated antibodies.

### Generation of *Cre*^*+/+*^*PTEN*^*fl/fl*^ MEFs

For generation of *PTEN*^*fl/fl*^*-ER-Cre* MEFs, *PTEN*^*fl/fl*^ mice were crossed with *CreER* mice, and E12.5 embryos were used to generate MEFs. *PTEN*^*fl/fl*^ mice and *CreER* mice were generously provided by Keunwook Lee (Hallym University, Chuncheon, Korea.). For deletion of the target gene, MEFs were plated at a density of 5 × 10^4^, then cultured in 0.5 μM 4-hydroxytamoxifen (4-OHT) containing culture media for 2 days.

### Statistical analysis

One-way ANOVA or *t*-tests with *P* < 0.05 were performed. Data are presented as the means ± SD.

## Electronic supplementary material

Supplementary figures and information
