# Supplementary material for: mTOR kinase leads to PTEN-loss-induced cellular senescence by phosphorylating p53
Source: Oncogene. 2018 Oct 18;38(10):1639–50. doi: 10.1038/s41388-018-0521-8 (PMC6755978; doi:10.1038/s41388-018-0521-8)
Supplement: Supplementary file 1 — Supplementary figures and information [file 41388_2018_521_MOESM1_ESM.pptx]

## Slide 1
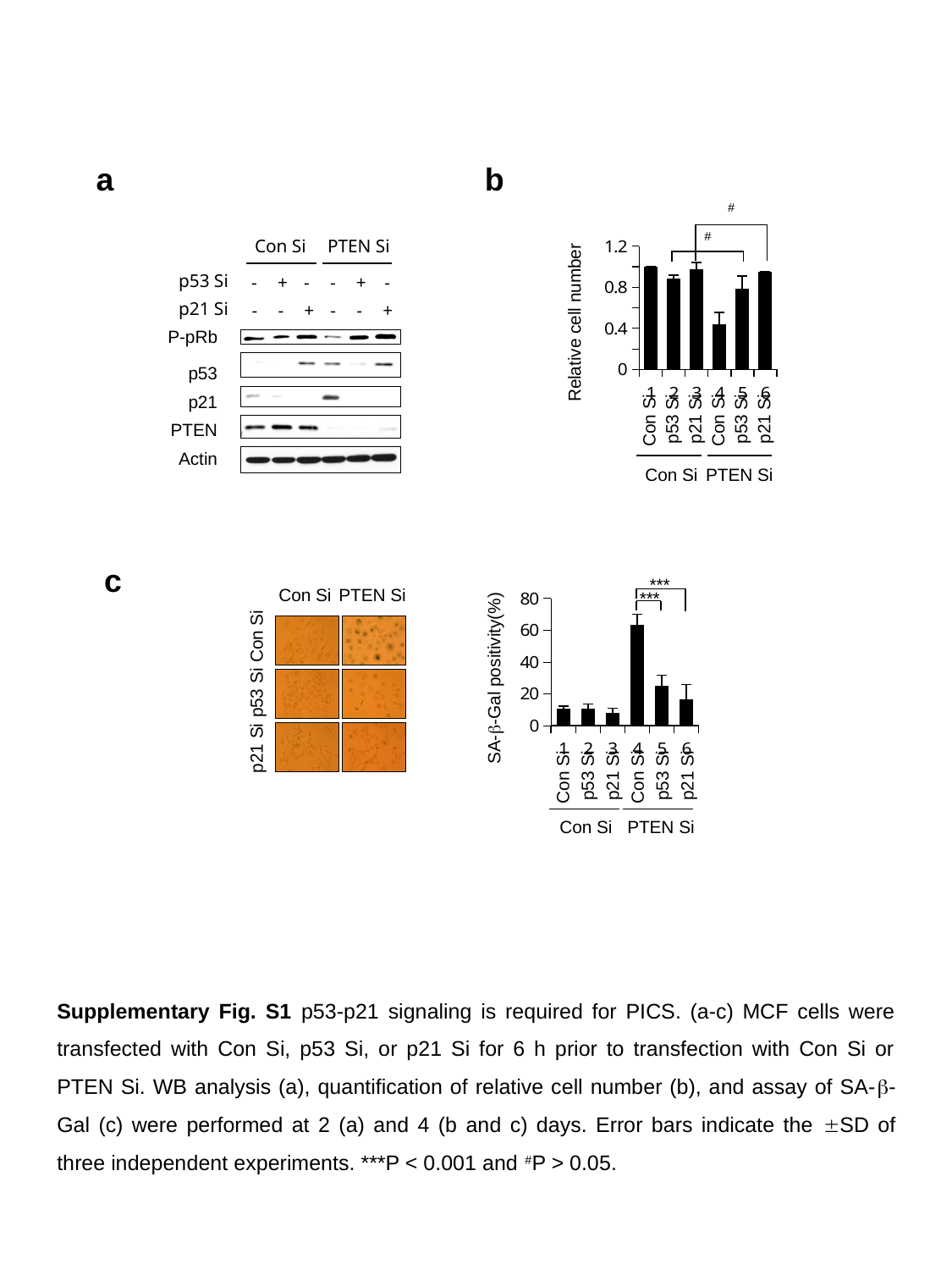

a
b
#
### Chart
| Category | |
|---|---|#
Con Si
PTEN Si
p53 Si
-
+
-
-
+
-
p21 Si
-
-
+
-
-
+
P-pRb
p53
p21
PTEN
Actin
Relative cell number
Con Si
p53 Si
p21 Si
Con Si
p53 Si
p21 Si
Con Si
PTEN Si
c
### Chart
| Category | |
|---|---|SA-β-Gal positivity(%)
Con Si
p53 Si
p21 Si
Con Si
p53 Si
p21 Si
Con Si
PTEN Si
***
***
Con Si
PTEN Si
Con Si
p53 Si
p21 Si
Supplementary Fig. S1 p53-p21 signaling is required for PICS. (a-c) MCF cells were transfected with Con Si, p53 Si, or p21 Si for 6 h prior to transfection with Con Si or PTEN Si. WB analysis (a), quantification of relative cell number (b), and assay of SA-β-Gal (c) were performed at 2 (a) and 4 (b and c) days. Error bars indicate the ±SD of three independent experiments. ***P < 0.001 and #P > 0.05.

## Slide 2
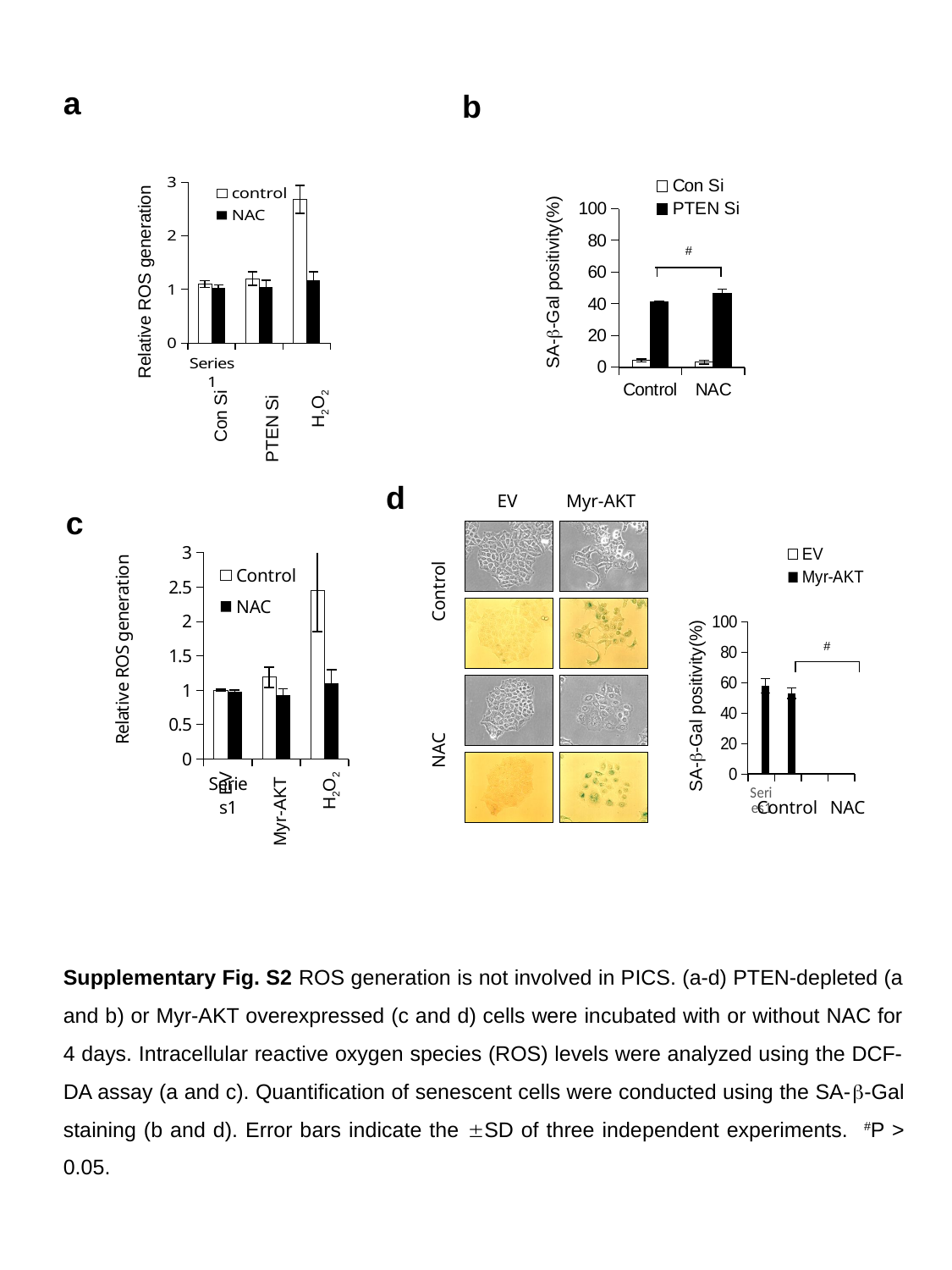

a
b
### Chart
| Category | control | NAC |
|---|---|---|
| | 1.1012658227848102 | 1.0253164556962024 |
| | 1.2025316455696202 | 1.0506329113924051 |
| | 2.680506329113924 | 1.1772151898734178 |Relative ROS generation
Con Si
H2O2
PTEN Si
### Chart
| Category | Con Si | PTEN Si |
|---|---|---|
| Control | 4.235140217480173 | 41.41385367706405 |
| NAC | 3.2222222222222228 | 46.41008107844095 |SA-β-Gal positivity(%)
#
d
### Chart
| Category | EV | Myr-AKT |
|---|---|---|
| | 6.3788019803757185 | 57.904169024712324 |
| | 5.963912630579298 | 53.0281313305049 |EV
Myr-AKT
Control
NAC
c
### Chart
| Category | Control | NAC |
|---|---|---|
| | 0.9999998359311181 | 0.9792451223551891 |
| | 1.191796359016184 | 0.9331007492861774 |
| | 2.445774824319143 | 1.1084493669484223 |EV
H2O2
Myr-AKT
#
SA-β-Gal positivity(%)
Control
NAC
Supplementary Fig. S2 ROS generation is not involved in PICS. (a-d) PTEN-depleted (a and b) or Myr-AKT overexpressed (c and d) cells were incubated with or without NAC for 4 days. Intracellular reactive oxygen species (ROS) levels were analyzed using the DCF-DA assay (a and c). Quantification of senescent cells were conducted using the SA-β-Gal staining (b and d). Error bars indicate the ±SD of three independent experiments. #P > 0.05.

## Slide 3
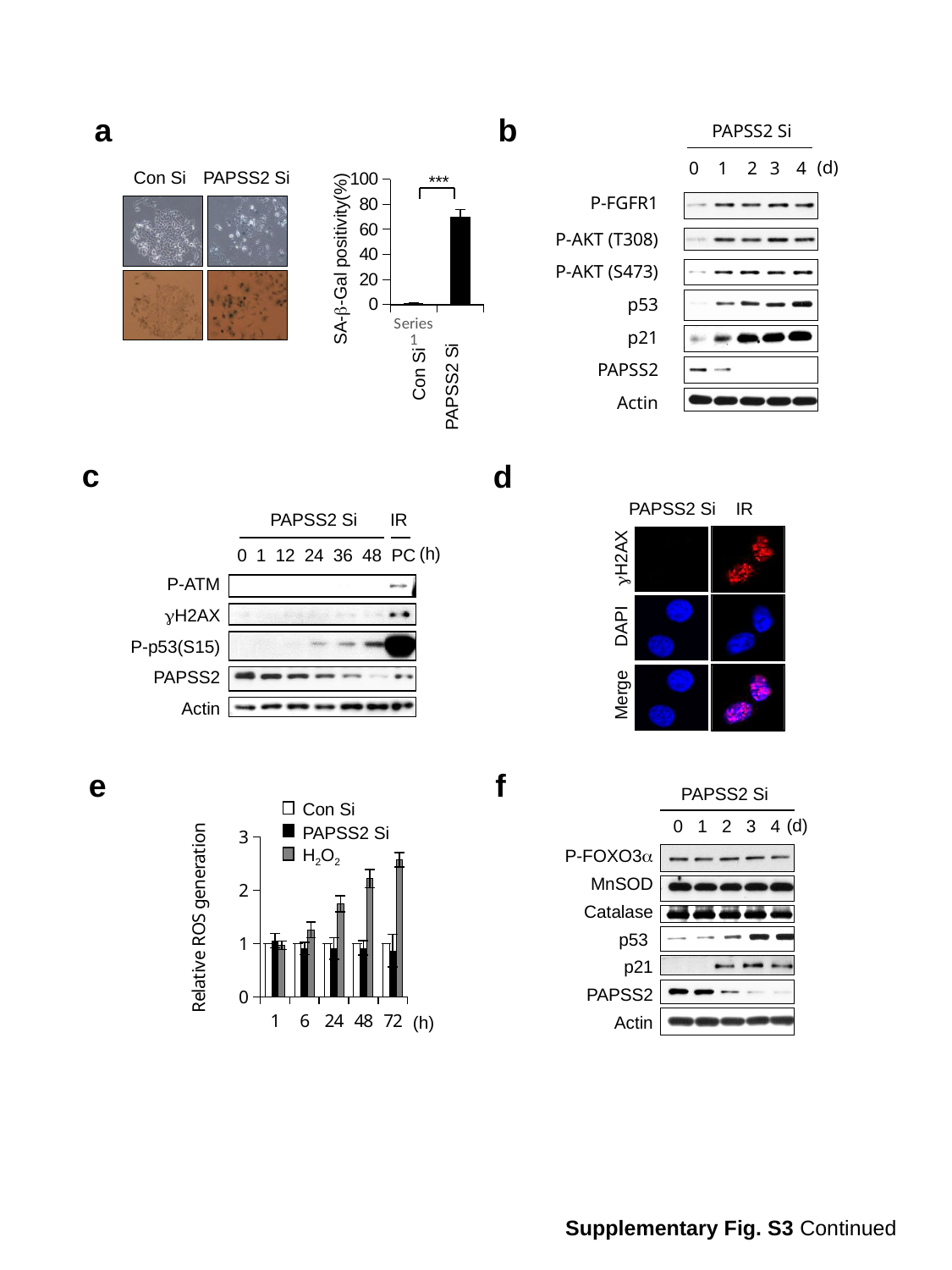

a
b
PAPSS2 Si
(d)
0
1
2
3
4
P-FGFR1
P-AKT (T308)
P-AKT (S473)
p53
p21
PAPSS2
Actin
Con Si
PAPSS2 Si
### Chart
| Category | |
|---|---|
| | 1.0497761912856254 |
| | 69.69135802469135 |
SA-β-Gal positivity(%)
Con Si
PAPSS2 Si
***
c
d
PAPSS2 Si
IR
γH2AX
DAPI
Merge
PAPSS2 Si
IR
(h)
0
1
12
24
36
48
PC
P-ATM
γH2AX
PAPSS2
Actin
P-p53(S15)
e
f
PAPSS2 Si
(d)
 0 1 2 3 4
P-FOXO3α
MnSOD
Catalase
p53
p21
PAPSS2
Actin
### Chart
| Category | Con Si | PAPSS2 Si | H2O2 |
|---|---|---|---|
| 1 | 1.0 | 1.0564055550038003 | 0.966549879092042 |
| 6 | 1.0 | 0.9091882020830576 | 1.2569094857904823 |
| 24 | 1.0 | 0.9058673506730667 | 1.7418581890324873 |
| 48 | 1.0 | 0.9171677256892617 | 2.2149060586887757 |
| 72 | 1.0 | 0.8673271503439978 | 2.5687962213649813 |Con Si
PAPSS2 Si
H2O2
(h)
 Supplementary Fig. S3 Continued

## Slide 4
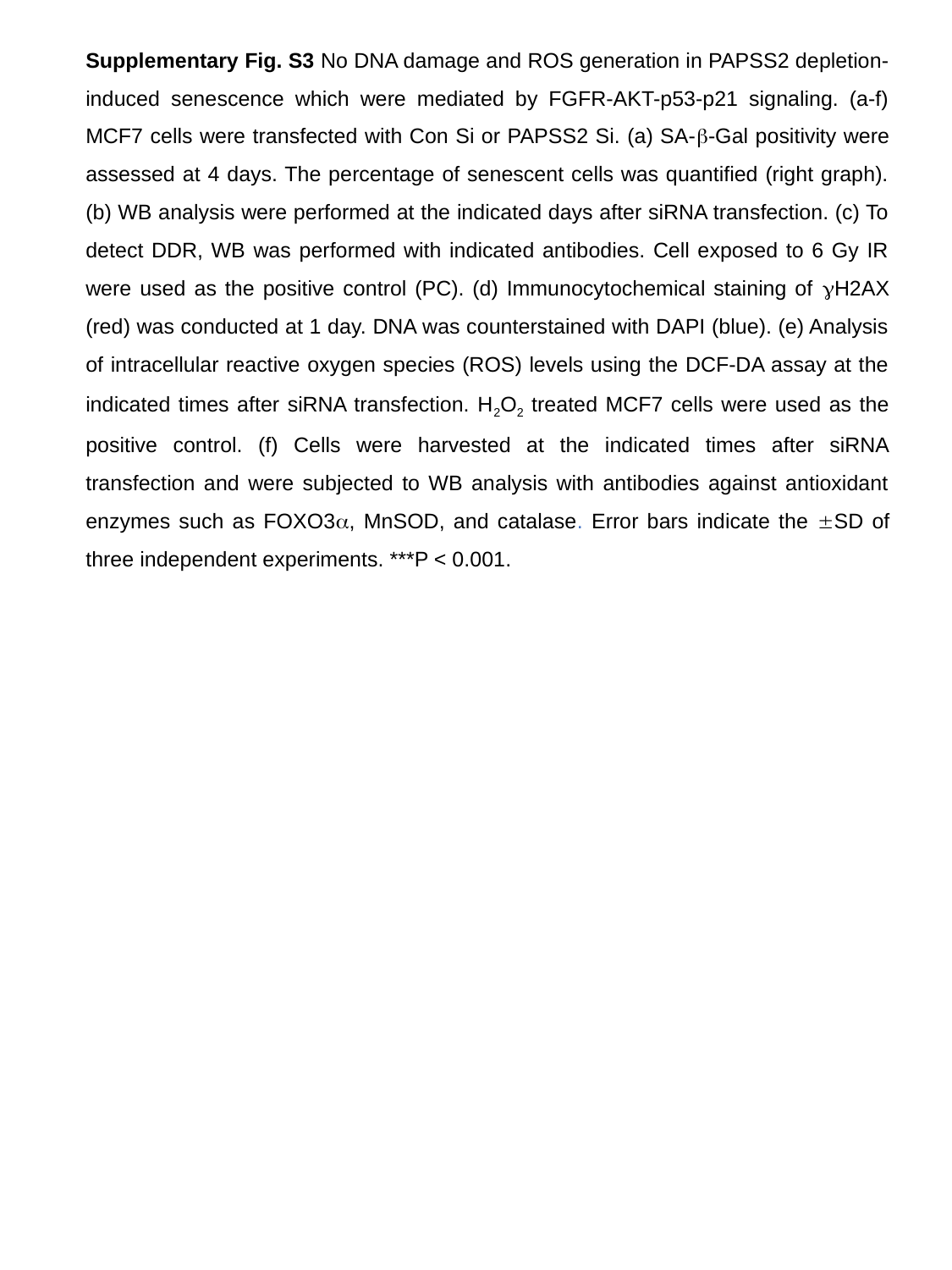

Supplementary Fig. S3 No DNA damage and ROS generation in PAPSS2 depletion-induced senescence which were mediated by FGFR-AKT-p53-p21 signaling. (a-f) MCF7 cells were transfected with Con Si or PAPSS2 Si. (a) SA-β-Gal positivity were assessed at 4 days. The percentage of senescent cells was quantified (right graph). (b) WB analysis were performed at the indicated days after siRNA transfection. (c) To detect DDR, WB was performed with indicated antibodies. Cell exposed to 6 Gy IR were used as the positive control (PC). (d) Immunocytochemical staining of γH2AX (red) was conducted at 1 day. DNA was counterstained with DAPI (blue). (e) Analysis of intracellular reactive oxygen species (ROS) levels using the DCF-DA assay at the indicated times after siRNA transfection. H2O2 treated MCF7 cells were used as the positive control. (f) Cells were harvested at the indicated times after siRNA transfection and were subjected to WB analysis with antibodies against antioxidant enzymes such as FOXO3α, MnSOD, and catalase. Error bars indicate the ±SD of three independent experiments. ***P < 0.001.

## Slide 5
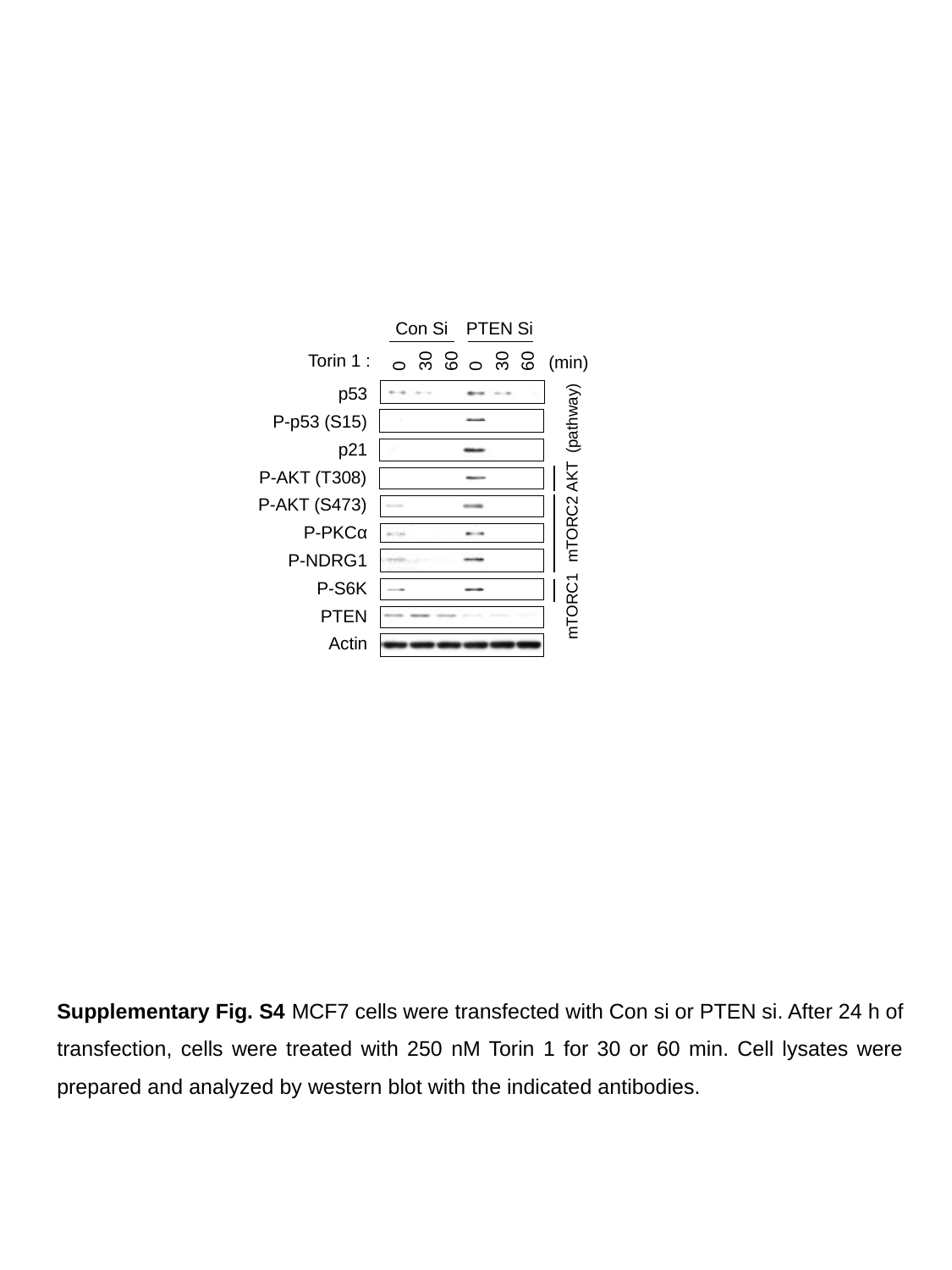

Con Si
PTEN Si
Torin 1 :
30
60
30
60
0
0
p53
P-p53 (S15)
p21
P-AKT (T308)
P-AKT (S473)
P-PKCα
P-NDRG1
P-S6K
PTEN
Actin
(min)
(pathway)
AKT
mTORC2
mTORC1
Supplementary Fig. S4 MCF7 cells were transfected with Con si or PTEN si. After 24 h of transfection, cells were treated with 250 nM Torin 1 for 30 or 60 min. Cell lysates were prepared and analyzed by western blot with the indicated antibodies.

## Slide 6
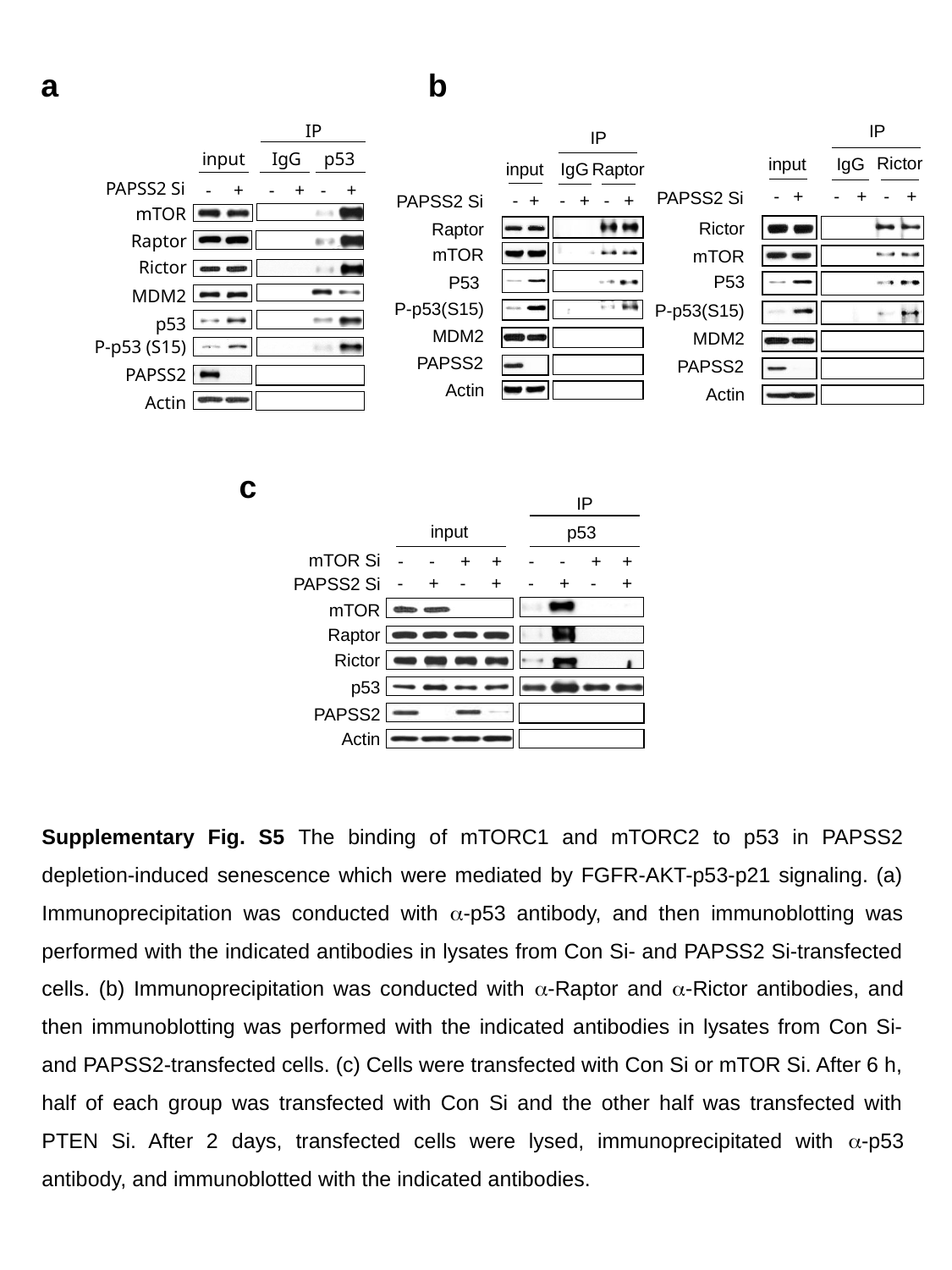

a
b
IP
Rictor
IgG
input
-
+
-
+
-
+
PAPSS2 Si
Rictor
mTOR
P53
P-p53(S15)
MDM2
PAPSS2
Actin
IP
input
IgG
p53
 PAPSS2 Si
-
+
-
+
-
+
mTOR
Raptor
Rictor
MDM2
p53
P-p53 (S15)
PAPSS2
Actin
IP
Raptor
IgG
input
-
+
-
+
-
+
PAPSS2 Si
Raptor
mTOR
P53
P-p53(S15)
MDM2
PAPSS2
Actin
c
IP
input
p53
mTOR Si
-
-
+
+
-
-
+
+
-
+
-
+
-
+
-
+
PAPSS2 Si
mTOR
Raptor
Rictor
p53
PAPSS2
Actin
Supplementary Fig. S5 The binding of mTORC1 and mTORC2 to p53 in PAPSS2 depletion-induced senescence which were mediated by FGFR-AKT-p53-p21 signaling. (a) Immunoprecipitation was conducted with α-p53 antibody, and then immunoblotting was performed with the indicated antibodies in lysates from Con Si- and PAPSS2 Si-transfected cells. (b) Immunoprecipitation was conducted with α-Raptor and α-Rictor antibodies, and then immunoblotting was performed with the indicated antibodies in lysates from Con Si- and PAPSS2-transfected cells. (c) Cells were transfected with Con Si or mTOR Si. After 6 h, half of each group was transfected with Con Si and the other half was transfected with PTEN Si. After 2 days, transfected cells were lysed, immunoprecipitated with α-p53 antibody, and immunoblotted with the indicated antibodies.

## Slide 7
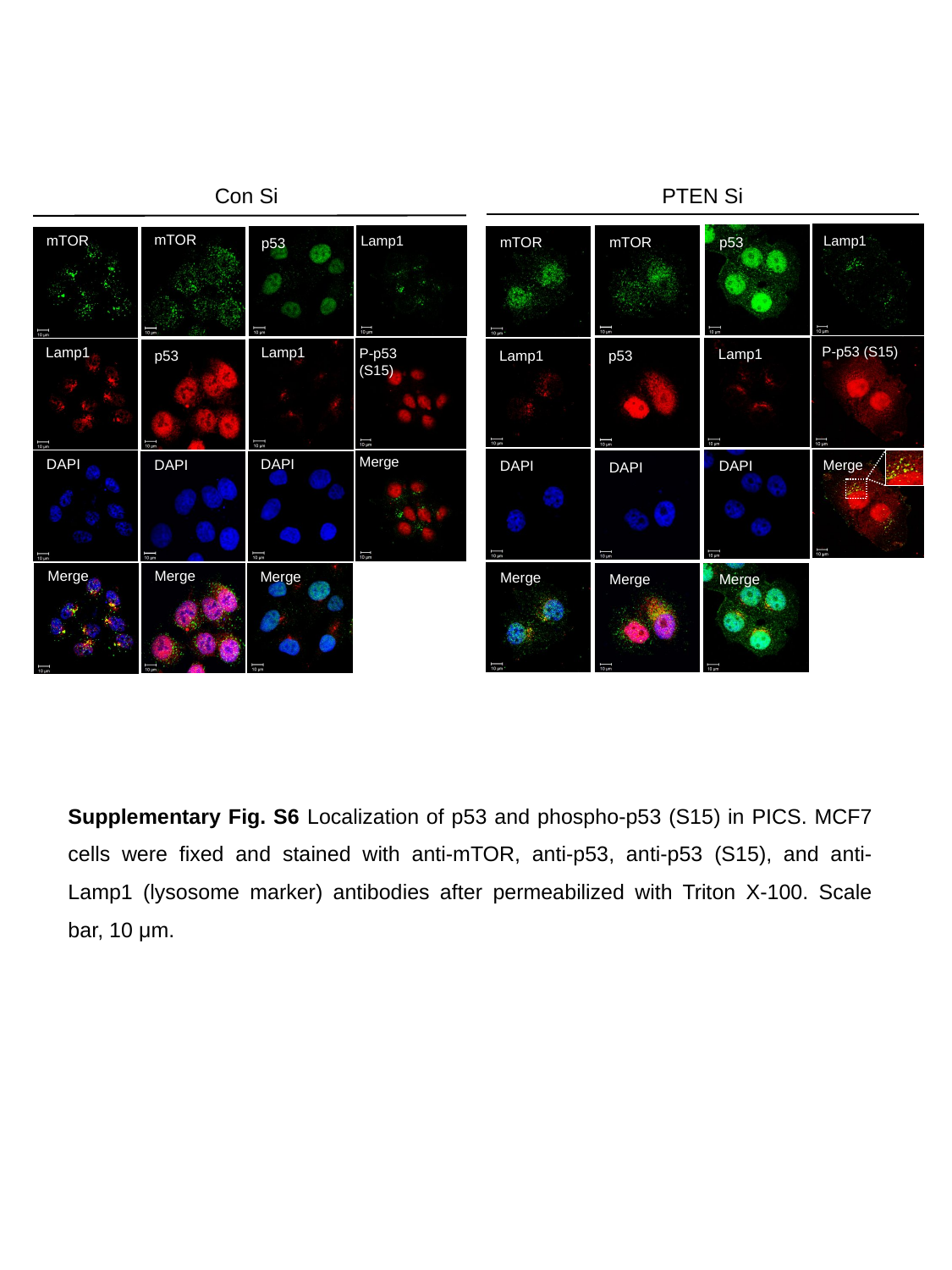

Con Si
PTEN Si
Lamp1
p53
mTOR
mTOR
P-p53 (S15)
Lamp1
p53
Lamp1
Merge
DAPI
DAPI
DAPI
Merge
Merge
Merge
mTOR
mTOR
p53
Lamp1
Lamp1
p53
DAPI
DAPI
DAPI
Merge
Merge
Merge
Lamp1
P-p53 (S15)
Merge
Supplementary Fig. S6 Localization of p53 and phospho-p53 (S15) in PICS. MCF7 cells were fixed and stained with anti-mTOR, anti-p53, anti-p53 (S15), and anti-Lamp1 (lysosome marker) antibodies after permeabilized with Triton X-100. Scale bar, 10 μm.

## Slide 8
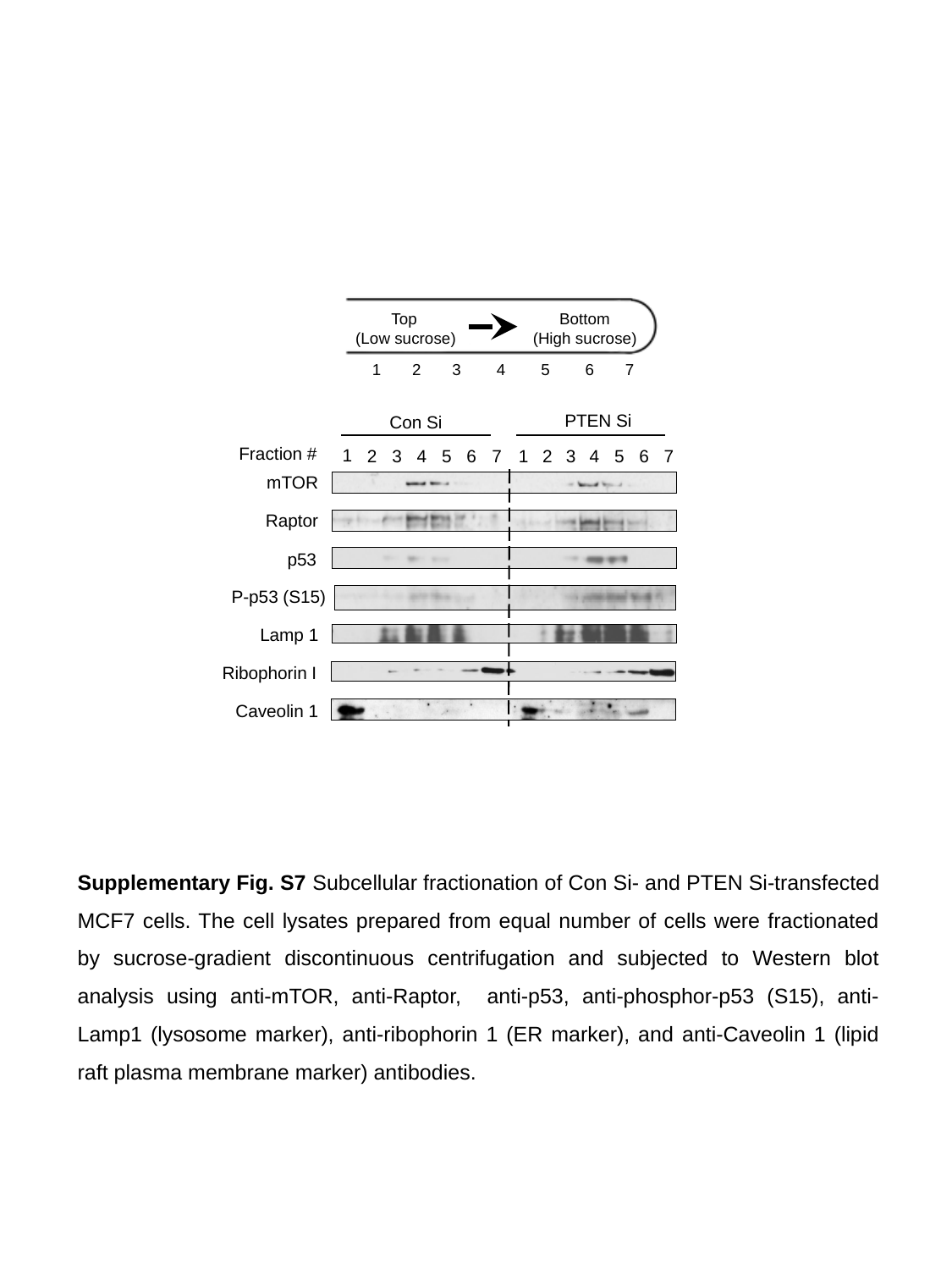

Top
(Low sucrose)
 Bottom
(High sucrose)
1 2 3 4 5 6 7
PTEN Si
Con Si
Fraction #
1
2
3
4
5
6
7
1
2
3
4
5
6
7
mTOR
Raptor
p53
P-p53 (S15)
Lamp 1
Ribophorin I
Caveolin 1
Supplementary Fig. S7 Subcellular fractionation of Con Si- and PTEN Si-transfected MCF7 cells. The cell lysates prepared from equal number of cells were fractionated by sucrose-gradient discontinuous centrifugation and subjected to Western blot analysis using anti-mTOR, anti-Raptor, anti-p53, anti-phosphor-p53 (S15), anti-Lamp1 (lysosome marker), anti-ribophorin 1 (ER marker), and anti-Caveolin 1 (lipid raft plasma membrane marker) antibodies.

## Slide 9
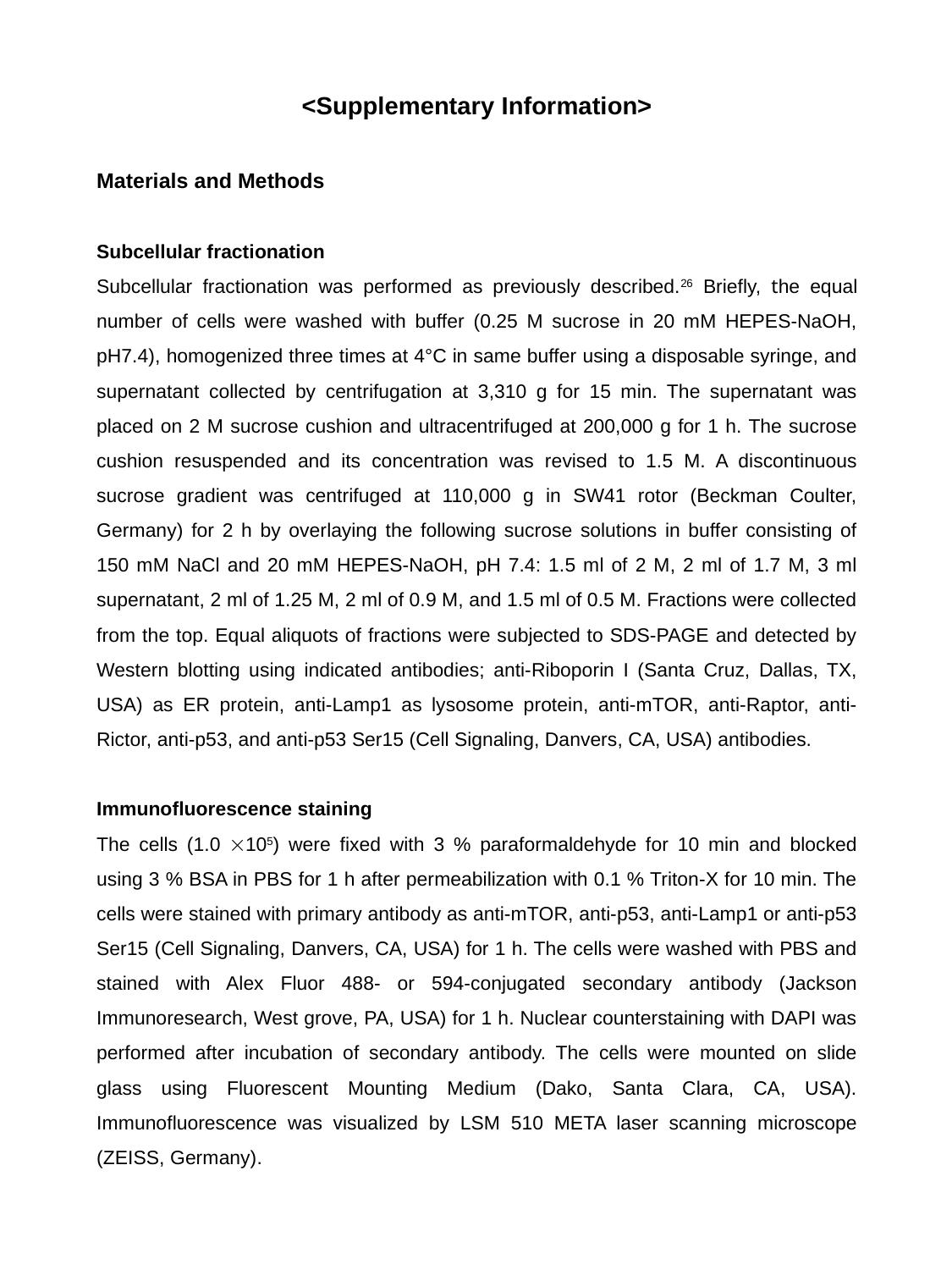

<Supplementary Information>
Materials and Methods
Subcellular fractionation
Subcellular fractionation was performed as previously described.26 Briefly, the equal number of cells were washed with buffer (0.25 M sucrose in 20 mM HEPES-NaOH, pH7.4), homogenized three times at 4°C in same buffer using a disposable syringe, and supernatant collected by centrifugation at 3,310 g for 15 min. The supernatant was placed on 2 M sucrose cushion and ultracentrifuged at 200,000 g for 1 h. The sucrose cushion resuspended and its concentration was revised to 1.5 M. A discontinuous sucrose gradient was centrifuged at 110,000 g in SW41 rotor (Beckman Coulter, Germany) for 2 h by overlaying the following sucrose solutions in buffer consisting of 150 mM NaCl and 20 mM HEPES-NaOH, pH 7.4: 1.5 ml of 2 M, 2 ml of 1.7 M, 3 ml supernatant, 2 ml of 1.25 M, 2 ml of 0.9 M, and 1.5 ml of 0.5 M. Fractions were collected from the top. Equal aliquots of fractions were subjected to SDS-PAGE and detected by Western blotting using indicated antibodies; anti-Riboporin I (Santa Cruz, Dallas, TX, USA) as ER protein, anti-Lamp1 as lysosome protein, anti-mTOR, anti-Raptor, anti-Rictor, anti-p53, and anti-p53 Ser15 (Cell Signaling, Danvers, CA, USA) antibodies.
Immunofluorescence staining
The cells (1.0 ×10^5^) were fixed with 3 % paraformaldehyde for 10 min and blocked using 3 % BSA in PBS for 1 h after permeabilization with 0.1 % Triton-X for 10 min. The cells were stained with primary antibody as anti-mTOR, anti-p53, anti-Lamp1 or anti-p53 Ser15 (Cell Signaling, Danvers, CA, USA) for 1 h. The cells were washed with PBS and stained with Alex Fluor 488- or 594-conjugated secondary antibody (Jackson Immunoresearch, West grove, PA, USA) for 1 h. Nuclear counterstaining with DAPI was performed after incubation of secondary antibody. The cells were mounted on slide glass using Fluorescent Mounting Medium (Dako, Santa Clara, CA, USA). Immunofluorescence was visualized by LSM 510 META laser scanning microscope (ZEISS, Germany).
